# Genetic and phenotypic links between obesity and extracellular vesicles

**DOI:** 10.1093/hmg/ddac069

**Published:** 2022-03-31

**Authors:** Ranran Zhai, Lu Pan, Zhijian Yang, Ting Li, Zheng Ning, Yudi Pawitan, James F Wilson, Di Wu, Xia Shen

**Affiliations:** Biostatistics Group, School of Life Sciences, Sun Yat-sen University, Guangzhou 510275, China; Center for Intelligent Medicine Research, Greater Bay Area Institute of Precision Medicine (Guangzhou), Fudan University, Guangzhou 511458, China; Department of Medical Epidemiology and Biostatistics, Karolinska Institutet, Stockholm 17177, Sweden; Biostatistics Group, School of Life Sciences, Sun Yat-sen University, Guangzhou 510275, China; Center for Intelligent Medicine Research, Greater Bay Area Institute of Precision Medicine (Guangzhou), Fudan University, Guangzhou 511458, China; Biostatistics Group, School of Life Sciences, Sun Yat-sen University, Guangzhou 510275, China; Center for Intelligent Medicine Research, Greater Bay Area Institute of Precision Medicine (Guangzhou), Fudan University, Guangzhou 511458, China; Department of Medical Epidemiology and Biostatistics, Karolinska Institutet, Stockholm 17177, Sweden; Department of Medical Epidemiology and Biostatistics, Karolinska Institutet, Stockholm 17177, Sweden; Centre for Global Health Research, Usher Institute of Population Health Sciences and Informatics, University of Edinburgh, Edinburgh EH8 9AG, UK; MRC Human Genetics Unit, Institute of Genetics and Molecular Medicine, University of Edinburgh, Edinburgh EH4 2XU, UK; Vesicode AB, Stockholm 17165, Sweden; Biostatistics Group, School of Life Sciences, Sun Yat-sen University, Guangzhou 510275, China; Center for Intelligent Medicine Research, Greater Bay Area Institute of Precision Medicine (Guangzhou), Fudan University, Guangzhou 511458, China; Department of Medical Epidemiology and Biostatistics, Karolinska Institutet, Stockholm 17177, Sweden; Centre for Global Health Research, Usher Institute of Population Health Sciences and Informatics, University of Edinburgh, Edinburgh EH8 9AG, UK; State Key Laboratory of Genetic Engineering, School of Life Sciences, Fudan University, Shanghai 200441, China

## Abstract

Obesity has a highly complex genetic architecture, making it difficult to understand the genetic mechanisms, despite the large number of discovered loci via genome-wide association studies (GWAS). Omics techniques have provided a better resolution to view this problem. As a proxy of cell-level biology, extracellular vesicles (EVs) are useful for studying cellular regulation of complex phenotypes such as obesity. Here, in a well-established Scottish cohort, we utilized a novel technology to detect surface proteins across millions of single EVs in each individual’s plasma sample. Integrating the results with established obesity GWAS, we inferred 78 types of EVs carrying one or two of 12 surface proteins to be associated with adiposity-related traits such as waist circumference. We then verified that particular EVs’ abundance is negatively correlated with body adiposity, while no association with lean body mass. We also revealed that genetic variants associated with protein-specific EVs capture 2–4-fold heritability enrichment for blood cholesterol levels. Our findings provide evidence that EVs with specific surface proteins have phenotypic and genetic links to obesity and blood lipids, respectively, guiding future EV biomarker research.

## Introduction

Overweight and obesity is currently major public health issue and most severe in high-income countries. In 2016, more than 1.9 billion adults were overweight, of those over 650 million were obese, and the prevalence of obesity continues to rise (1). Obesity is a risk factor for various chronic diseases such as type 2 diabetes (2) and cardiovascular disease (3), which in turn can lead to reduced quality of life, premature death or disability (4). Obesity is defined as an excessive accumulation of body fat and is most estimated using body mass index (BMI, kg/m^2^) in epidemiological studies and clinical practice. However, BMI cannot distinguish lean from fat mass, nor does it capture information on where in the body the adiposity is concentrated. Abdominal fat and visceral fat (fat around the internal organs of the body) in particular, has been linked with obesity-related metabolic risk factors, including high total and low-density lipoprotein cholesterol (HDL-c and LDL-c) (5,6), while fat in the lower body (e.g. around the hips) is associated with a protective lipid and glucose profile (7). Thus, proxies of abdominal adiposity, like waist circumference (WC) and waist-to-hip ratio (WHR), are of increasing interest in investigating the mechanism and regulation of obesity and its complications.

Genome-wide association studies (GWAS) have begun to elucidate the genetic architecture of body fat distribution phenotypes. Hundreds of loci associated with WC and WHR have been identified (8–10). However, the underlying biological knowledge gained from GWAS-discovered single nucleotide polymorphisms (SNPs) is, so far, limited. The discovered SNPs have tiny effects, explaining a small proportion of phenotypic variance of these abdominal adiposity-related phenotypes (11). Efforts have been made to integrate GWAS summary statistics with gene expression in adipose tissues to further characterize obesity-causal genes (12). Integration of GWAS and gene expression quantitative trait loci (eQTL) studies has also achieved success in prioritizing genes in obesity-related pathways (13,14).

To better understand the regulation of abdominal adiposity, adiposity-related biomarkers are also of general interest. In the last decade, extracellular vesicles (EVs) have become essential in biomarker discovery for their emerging roles in physiological and pathological pathways. These nanometer-sized membranous vesicles emitted from nearly all cells have a variety of functions and can regulate target cells’ metabolism by conveying genomic material (15) and proteins (16). Recent studies reported that circulating EVs were elevated in obese humans (17), and levels of EVs and specific proteins in EVs were altered in diabetic mice (18). In these studies, EVs were isolated using ‘EV-specific’ markers in bulk, considering the level of EVs or a protein carried by the EVs as an overall measurement. Recently, a new technology named proximity barcoding assay (PBA) was developed to detect multiple surface proteins on single EVs in a high-throughput manner (19). This allows us to quantify EVs with specific surface markers, thus better understanding the heterogeneity of EVs.

Attempting to link specific EVs to obesity, in this study, we aim to identify particular types of EVs associated with abdominal adiposity. We approach this by investigating EVs with specific adiposity-related surface proteins based on established GWAS summary statistics for adiposity-related phenotypes, followed by validation using individuals’ body fat distribution measurements. We perform genomic analysis for SNPs associated with the levels of specific EVs. We show that the heritability for plasma LDL-c and total cholesterol levels are enriched at the loci associated with EVs carrying adiposity-related surface proteins.

## Results

### Prioritizing obesity-related EV surface markers using GWAS summary statistics

**Figure 1:**
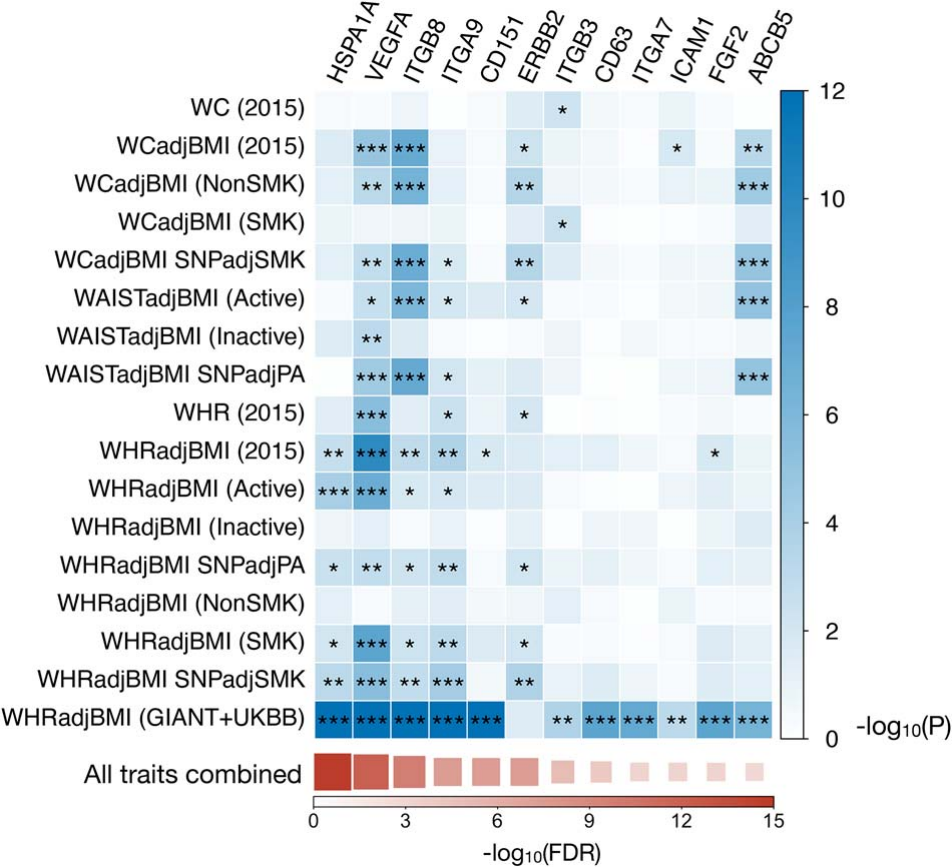
Coding genes for the measured EV proteins that are associated with obesity-related traits. We performed multi-trait genome association analysis using summary association statistics of 17 traits from the GIANT consortium, which resulted in 18 (17 univariate and a multivariate analyses) association *P*-values for each gene. Only the genes with FDR_Multivariate_ < 0.01 are shown and ordered by −log10(FDR_Multivariate_) value in descending order. For the association with each trait, −log10(*P*) values are colored in blue, and the associations with FDR < 0.05, 0.01 and 0.001 are marked by ^*^, ^**^ and ^***^, respectively.

The commercial EV surface marker panel available to us consists of 113 proteins targeted by specific antibodies (see section Materials and Methods). To identify the associations between the encoding genes of these protein markers and obesity, we started by constructing corresponding gene-level association scores for obesity-related phenotypes. We collected the established summary-level association data for WC and WHR related phenotypes conducted by the Genetic Investigation of Anthropometric Traits (GIANT) consortium. Those traits include WC and WHR, as well as WC and WHR adjusted or stratified by other factors (BMI, smoking, and physical activity), all using European subjects (details are in Supplementary Material, Table S1). We also performed a multi-trait GWAS analysis of those summary statistic data using MultiABEL (20), where we can get combined SNP *P*-values of those traits. We then used the Pathway Scoring Algorithm (PASCAL) to derive gene-level *P*-values from SNP-level association statistics (21). Out of the 113 coding genes, 12 were significantly associated with multi-trait waist adiposity GWAS (false discovery rate (FDR) < 0.01) (Fig. 1).

### Measurement quality of candidate EV protein markers

**Figure 2:**
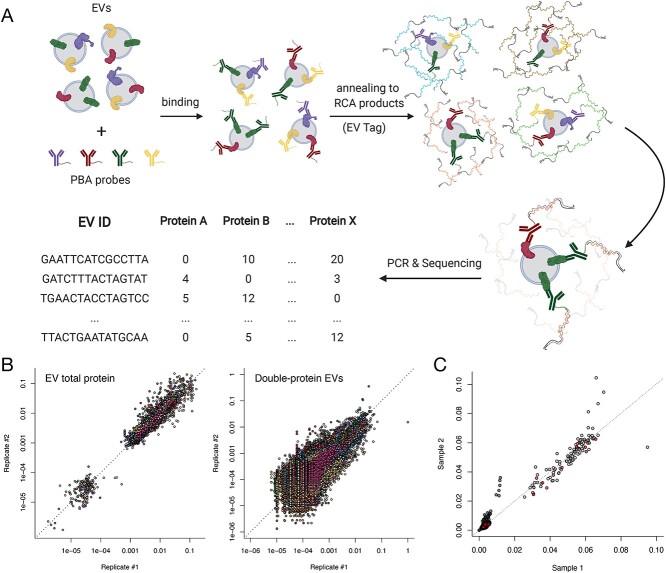
Identifying surface proteomics profile of extracellular vesicles. (**A**) Schematic representation of proximity barcoding assay (PBA). EVs are firstly incubated with PBA probes (containing a protein tag), then each EV is allowed to hybridize with a unique RCA product (including a unique EV tag), followed by enzymatic extension, protein tag on the same EV is then incorporated with its EV tag, which can be sequenced after polymerase chain reaction. From the final pool of reads, the surface proteins are quantified for millions of individual EVs. (**B**) EV proteomic profile of two independent testing individuals. Percentages of EVs that carry each single protein and double-protein combination are robustly measured and consistent within the two independent samples. (**C**) EV proteomic profile of 96 ORCADES samples from two experiments (replicates 1 and 2). Each point stands for a specific type of EV (left panel for the abundance of EVs with at least one kind of protein, and the right panel for the abundance of EVs with at least two kinds of proteins). Different samples are color-coded.

The PBA technique is briefly described in Figure 2A (see section Materials and Methods). Using the PBA technique, we could detect the abundance of the 113 surface proteins across many single EVs in each plasma sample. Before measuring multiple individual samples, we performed in-depth sequencing quantification (50 million reads per sample) for two independent plasma test samples from two unrelated individuals. We obtained approximately 1.5 million EVs in each of two testing plasma samples, of which ~62% were singleton (EVs that only carry one type of protein) (Supplementary Material, Fig. S1A). To reduce background noise, we conducted data quality-control procedures (see section Materials and Methods), which resulted in 61 108 EVs measured with high quality in sample 1 and 73 139 in sample 2.

Most of these EVs (95.6% and 96.4% in samples 1 and 2) carry 1 to 10 different proteins, and the most abundant EVs carry 5–6 different proteins (Supplementary Material, Fig. S1B). Due to multiplex limitations, especially when sequencing depth is not as high when measuring many samples, single EVs carrying many overlapping proteins are challenging to quantify in two independent samples. We thus focused on investigating EVs containing one or two of the obesity-related markers identified above across possible proteins and protein–protein combinations. The abundance of such EVs with specific marker profiles was consistently measured both between samples (Fig. 2B) and replicates (Fig. 2C), suggesting the stability of PBA in measuring these protein markers.

### Validating the association between overall protein levels and obesity

To validate the obesity-related EV surface markers prioritized by GWAS association statistics, we conducted the 113-marker single-EV surface protein quantification using PBA in 96 selected individuals in the ORCADES cohort (see section Materials and Methods). As a comparison, we also quantified the total abundance of 37 proteins out of the 113 that could be measured in plasma by the Olink Proximity Extension Assay (PEA) panels. For these individuals in the cohort, besides general anthropometric measurements such as height, BMI, WHR, and WC, we also had Dual Energy X-ray Absorptiometry (DEXA) scan data available to quantify their fat distribution in the body. The DEXA phenotypes included total lean mass, trunk fat, trunk lean mass and visceral fat.

**Figure 3:**
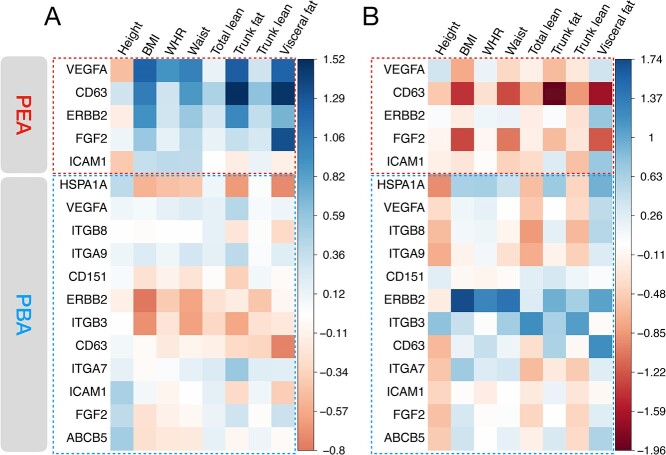
Associations between total protein level and anthropometric measurements in 96 ORCADES samples. In **A** and **B**, the upper part shows the effect of total protein level measured by PEA (Olink panels), and the lower part shows the effect of total protein quantified by PBA. Proteins in red dotted boxes are the markers of interest that overlap with Olink proteins. (**A**) The main effect of total protein count on seven body fat distribution-related traits, adjusted for sex and age. (**B**) Sex-by-protein interaction effect. Males are coded as 1 while females as 0, i.e. positive interaction (in blue) represents that the overall protein effects are weaker in men than in women. The effects (Beta) are color-coded.

First, we tested the associations between adiposity phenotypes and overall protein levels quantified by both PBA and PEA (Fig. 3A; see section Materials and Methods). Protein abundance on the EV surface had little association with the obesity-related phenotypes in our 113-marker panel. While for two proteins quantified by PEA, levels of VEGFA and CD63 in plasma were associated with body fat distribution-related traits. Among those associations, levels of plasma CD63 have the strongest effect on trunk fat mass (effect size = 1.25, equivalent to 6.5 standard deviation increase of trunk fat per standard deviation of plasma CD63 level, *R*^2^ = 0.13, *P* = 0.011). There is a positive correlation between plasma VEGFA level and BMI, supported by another study (22) where they found a positive correlation between circulating VEGF levels and BMI. We then tested the sex-by-protein interaction effect on those phenotypes (Fig. 3B). Although plasma protein levels showed almost no interaction effects, the overall EV protein levels, especially that of ERBB2, had significant interaction effects on fat distribution traits such as waist circumference: EV ERBB2 levels are more strongly associated with abdominal adiposity in women.

### Validating the association between marker-specific EVs and obesity

**Figure 4:**
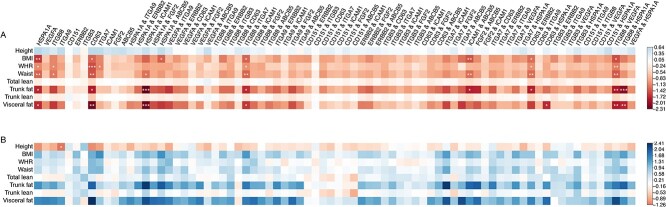
Association between the abundance of specific EVs and anthropometric measurements in 96 ORCADES samples. (**A**) The main effect of the abundance of specific EVs on seven body fat distribution-related traits, adjusted for sex and age. (**B**) Sex-by-EV interaction effect. Males are coded as 1 while females as 0, i.e. positive interaction (in blue) represents that the overall EV effects are weaker in men than in women. The effects (Beta) are color-coded; Significance with overall FDR thresholds of 0.05, 0.1 and 0.2 are marked by ^***^, ^**^ and ^*^.

As well as the overall level of protein on the surface of EVs, the PBA technology can also provide us with surface protein profiles for individual EVs. Here, we tested the associations between the obesity-related phenotypes and the levels of EVs with specific protein profiles (Fig. 4A; see section Materials and Methods). In contrast to overall protein levels on the EV surface, levels of EVs with single-marker specificity showed strong associations with these phenotypes. To be more stringent, we performed FDR calculation across all the statistical tests for the associations regarding total protein levels and specific EVs (*P*-value distribution of each part of the analysis is given in Supplementary Material, Fig. S2). Levels of EVs carrying HSPA1A (EV_HSPA1A_) had the most significant association with BMI (effect size = −1.62, equivalent to 7.60 standard deviation decrease of BMI per standard deviation of plasma EV_HSPA1A_ level, *R*^2^ = 0.17, *P* = 4.5$\times$10^−4^), meaning that individuals with less of these EVs have significantly higher BMI. Focusing on EVs identified by two protein markers on their surface (double-marker specificity) revealed even stronger associations, for EVs carrying several specific protein–protein combinations had even stronger associations. For instance, levels of EV_HSPA1A & ICAM1_ had the strongest association with trunk fat (effect size = −2.31, equivalent to 12.0 standard deviation decrease of trunk fat per standard deviation of plasma EV_HSPA1A & ICAM1_ level, *R*^2^ = 0.27, *P* = 7.1 × 10^−5^) and visceral fat (effect size = −2.22, equivalent to 0.70 standard deviation decrease of visceral fat per standard deviation of plasma EV_HSPA1A & ICAM1_ level, *R*^2^ = 0.31, *P* = 1.1 × 10^−4^). These are stronger associations than observed for these traits with EV_HSPA1A_, but levels of EV_HSPA1A & ICAM1_ were less significantly associated with BMI. Similarly, EV_ITGB8_ abundances were not a better indicator of adiposity than its sub-population EV_ITGB8 & VEGFA_ or EV_ITGB8 & ITGA7_.

These associations were observed with body fat-related traits, like the trunk and visceral fat, but not with total or trunk lean mass. We also tested the sex-by-EV interaction effects on these phenotypes (Fig. 4B). The above significant types of EVs tended to have different effects in different sexes. Although the signals were not statistically robust, the results also suggested stronger sex-by-EV interaction effects in women than in men.

### EV-associated SNPs capture enriched heritability for blood lipids

Although with limited power, we performed 12 × (113—12) + 66 = 1276 GWAS of double-protein EV (EV carrying at least one of 12 markers) levels in 96 ORCADES individuals. For each double-protein EV phenotype, we tested the associations of genome-wide SNPs imputed to the 1000 Genomes reference panel (minor allele frequency > 0.2 to avoid inflated false positives due to the small sample size) using a linear model, adjusted for sex, age, population stratification and other covariates (see section Materials and Methods). Despite the small sample size, such GWAS resulted in an association *P*-value distribution significantly deviating from the null (Supplementary Material, Fig. S3). With a stringent minor allele frequency cutoff and a Bonferroni-corrected significance threshold of *P* < 5 × 10^−8^/1276 = 3.9 × 10^−11^, we did not report any specific SNPs associated with the EV phenotypes.

**Figure 5:**
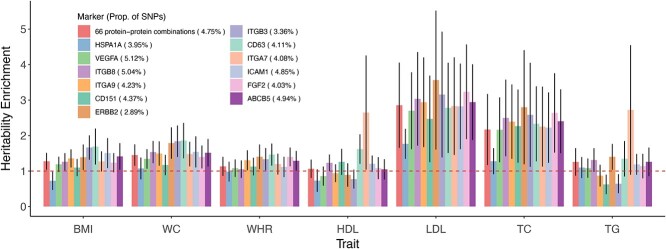
Heritability enrichment of EV-associated SNPs. Heritability enrichment estimation of EV-associated SNPs for each protein marker. Each bar represents heritability enrichment on an obesity-related trait, and the error bars represent standard errors. The red dashed line indicates no enrichment.

Nevertheless, to understand the role of the EV-associated SNPs in the regulation of obesity, we used stratified LD score regression (23) (S-LDSC) to evaluate heritability enrichment on these SNPs. Seven traits were considered, including BMI, WC, WHR, and four classical blood lipids (24) (high- and low-density lipoprotein cholesterol (HDL-c, LDL-c), total cholesterol, and triglycerides). Using a less stringent threshold in the EV GWAS (*P* < 1 × 10^−6^), we annotated the SNPs associated with EVs carrying different types of protein–protein combinations (see section Materials and Methods). Heritability enrichment was detected for most annotations. Specifically, we found 2–4-fold heritability enrichment for LDL-c and total cholesterol that is positively correlated with abdominal fat (Fig. 5). These results indicated that the EVs with particular surface proteins are not only phenotypically but also genetically correlated with obesity and cholesterol metabolism.

## Discussion

In this study, we investigated the association between plasma EV levels and body fat distribution, with an emphasis on abdominal fat. We found that the levels of plasma EVs carrying specific surface proteins were negatively associated with adiposity-related measurements, including BMI, WC, WHR, trunk fat and visceral fat. For some types of EV (e.g. EV_HSPA1A & ICAM1_), the association with trunk and visceral fat mass is more significant than BMI, WC, or WHR, implying that those EVs might be more related to or arise from visceral adipose tissue. Notwithstanding our inability to track the tissue or even cell-type origins of these EVs, to our knowledge, our results for the first time demonstrate that protein-specific EVs in plasma are associated with body fat distribution and obesity in humans.

We were able to identify a few genome-wide significant SNPs associated with certain EVs with specific surface protein markers, despite that the small sample size limited our discovery power. The power of the EV-QTL scan might come from two sources: (1) The PBA technique allowed screening across EVs with many different protein–protein combinations. (2) The double-protein signature on the EVs enhanced the measurement specificity so that a more specific genetic basis for the EVs could be mapped. However, due to the lack of replication, such perspectives remain to be validated in future larger GWAS analyses of these EV phenotypes.

We integrated GWAS summary-level data with EV protein measurements. Still, our current sample size is insufficient to conduct systematic genomic analyses of EV surface proteins and all types of protein-specific EVs. In future studies, we foresee that expansion in sample size would open a new area for extracellular vesicle omics, providing power to establish the genetic architecture of various kinds of EVs. Such resources would allow better causal inference of EVs on disease phenotypes. This is particularly possible for circulating EVs, for which only plasma samples would be required.

Although measured from the blood, with tissue- or cell-type-specific protein markers designed in the PBA panel, one can further consider PBA as a proxy single-cell technology. Future expansion of our panels may allow us to investigate specific tissues, either via tissue-specific markers or deconvolution algorithms, when tissue-specific EV proteomic profiles become available. This could expand this exciting research area to explore the genetic basis of tissue- and cell-type-specific EVs.

Although it is not novel to identify different obesity regulatory mechanisms in men and women, it is also noteworthy that we detected a more substantial sex-by-EV interaction effect in women than in men, meaning EVs have more impact on women in terms of their association with body fat. While sex differences in obesity and body fat distribution are well established, our results suggest that EVs might also play a role in these differences. Our results also reveal that EV-QTLs share heritability with LDL-c and total cholesterol, both of which increase the risk of cardiovascular and metabolic diseases. Though the biological mechanism underlying such associations remains unknown, our results show genetic connections between levels of protein-specific EVs and adiposity measurements.

A recent study showed that the liver could secret EVs to modulate adipocyte remodeling in response to excessive lipid (25). It is well known that RNA transferred by EVs can regulate or serve as the template for protein synthesis in the recipient cells (26). Another study has shown that miRNAs in exosomes (a subpopulation of EVs) from obese visceral adipocytes could down-regulate the expression of ACVR2B in the TGF-$\beta$ signaling pathway (27), which plays a crucial role in obesity and insulin resistance (28–30). Such discoveries are consistent with our finding that individuals with higher visceral fat tend to have fewer EVs with ITGB8 & VEGFA, where ITGB8 can activate TGF-$\beta$ when combined with ITGAV (integrin $\alpha$ subunit V) (31,32).

The heterogeneity of the EV population has been problematic in studies using EVs as a diagnostic and therapeutic biomarker. Kaur *et al.* (33) reported that EVs captured by different surface protein markers had distinct RNA profiles. In accordance with their study, our results showed that EVs categorized by different surface proteins, such as integrins (ITGB8, ITGA9, and ITGA7), tetraspanins (CD63), heat shock protein (HSPA1A), etc., have similar but varied associations with human adiposity traits. Such results highlighted the heterogeneity of EV subpopulations, suggesting that single-EV measurement technologies such as PBA have the potential to study the origins and functions of different types of EVs.

We also provided a piece of new evidence on the potential EV-based liquid biopsy as a tool. The PBA technique only focuses on the membranous proteins of EVs at present, neglecting other functional molecules such as miRNAs, lipids, and endogenous proteins (34). It limits our insight into the underlying regulatory mechanism of EVs in physiological and pathological pathways. However, being able to quantify the particular molecular profiles of single EVs has already brought substantial power.

## Materials and Methods

### ORCADES samples

Our 96 individuals are a subset of 2080 volunteers from the Orkney Complex Disease Study (ORCADES) cohort. ORCADES is a family-based, cross-sectional study that seeks to identify genetic factors influencing cardiovascular and other disease risk in the isolated archipelago of the Orkney Isles in northern Scotland (35). Genetic diversity in this population is decreased compared to Mainland Scotland, consistent with the high levels of endogamy historically. About, 2078 participants aged 16–100 years were recruited between 2005 and 2011, most having three or four grandparents from Orkney, the remainder with two Orcadian grandparents. Fasting blood samples were collected, and many health-related phenotypes and environmental exposures were measured in each individual. All participants gave written informed consent, and the study was approved by Research Ethics Committees in Orkney and Aberdeen (North of Scotland REC). Genome-wide genotyping was performed using Illumina HumanHap300 and OmniExpress arrays. The individuals were randomly selected with a balanced sex ratio from the subset of individuals with the most measured phenotypes.

### Profiling EV surface proteins with PBA

All the samples were sent to Vesicode AB (Sweden) with dry ice, and proximity barcoding assay (PBA) (19) was carried out according to Vesicode AB’s PBA standard operation procedure (SOP). The raw data BCL sequencing files were converted to a fastq file with bcl2fastq software (Illumina). Proteins were determined by mapping protein tags in the sequences to the designed panel of oligonucleotides conjugated to antibodies. The EV tags were extracted and used as the identity for single EVs. A file consisting of the EV tags and their protein expression profile was obtained for each sample. Different samples resulted in a different number of sequenced reads; each read count of the protein expression was normalized by the total number of reads in the corresponding sample. The readouts were comparable across samples as ‘the proportion of proteins per sample.’

### EV profiles of two testing samples via deep sequencing

Using deep sequence quantification (50 million per sample), we obtained approximately 1.5 million EVs in each of two testing plasma samples, of which ~62% were singleton (EVs that only carry one type of protein), where ~50% of these only carry one protein. To reduce background noise, we first filtered EVs that have more than five proteins, which resulted in our EV matrix (*D*, $m\times n$). We calculated the expected values and *c* values of *D*, using the following equation: $E=A{B}^T$where *A* is the matrix of sums of rows ($1\times m$), and *B* is the matrix of sums of columns ($1\times n$), $\chi =(D-E)/\sqrt{E}$ cells with top 95% quantile chi values were considered to be actual protein count, others were reset to be 0.

### Gene-based statistics from GWAS summary statistics

Gene-based *P*-values were generated by PASCAL (21). In our analysis, the window size for computing sum and maximum of chi-squared statistics was 50 kb up- and downstream. For better quality, the LD information was obtained from the UK10K data (https://www.ebi.ac.uk/ega/datasets/EGAD00001000776) instead of the default 1000 Genomes project.

### Normalization of EV phenotypes and regression models

We used 96 individuals from the ORCADES cohort to test the associations between obesity-related traits and the abundance of EVs with specific surface proteins markers. For each obesity-related trait $Y$, we standardized the data as ${Y}^{\ast }=(Y-\mu )/\sigma$, where $\mu$ and $\sigma$ represent the mean and standard error of the phenotypic data vector. For the abundance of each EV phenotype $X$, corresponding to a type of EVs that carry a particular protein profile, we inverse-Gaussian transformed the data vector into ${X}^{\ast }$, following a standard normal distribution. We conducted a multiple linear regression model to test specific associations between EV types and the obesity-related traits:

$$ {Y}^{\ast }=\mu +{X}^{\ast }\ {\beta}_{\mathrm{EV}}+\mathrm{Sex}\ {\beta}_{\mathrm{Sex}}+\mathrm{Age}\ {\beta}_{\mathrm{Age}}+{X}^{\ast}\mathrm{Sex}{\beta}_{\mathrm{EV}-\mathrm{by}-\mathrm{sex}}+\varepsilon $$

where $\mu$ is the overall mean, $\beta$’s are the corresponding effect parameters, and $\varepsilon$ is the residual. The regression analysis was performed using the lm() procedure in *R*.

### Genome-wide association analysis of protein-specific EVs

Prior to GWAS, each EV phenotype was adjusted for fixed effects of sex, age, and the other experimental factors. The residuals were inverse-Gaussian-transformed to standard normal distributions. The residuals expressed as *Z*-scores were used for all genetic association analyses. In both the genotypes from the SNP array and 1000 Genomes-imputed data, markers with minor allele frequency < 0.05 were excluded. Population structure was corrected using the GRAMMAR+ transformation (36), implemented in the GenABEL package (37). The genomic relationship matrix used in the analyses was generated by the ibs() function (with weight = ‘freq’ option), which uses SNP array data to estimate the realized pairwise kinship coefficient. The polygenic() function was used to obtain the GRAMMAR+ transformed phenotypes (grresidualY) from linear mixed models. All univariate GWAS inflation factors (lambda values) were close to 1, showing that this method efficiently accounts for family structure. The processed phenotypes were tested in genome scans using REGSCAN (38).

### Heritability enrichment analysis

For each obesity-related protein marker, we had 112 GWASed EV phenotypes for the EVs that carry different protein–protein combinations with this particular protein. To overcome the power loss due to the small sample size, for each of the 12 obesity-related proteins, we extracted all the SNPs with *P* < 1 × 10^−6^ in all 112 GWAS. In addition, we also extracted all the SNPs with *P* < 1 × 10^−6^ in 66 GWAS for the EVs that carry obesity-related protein–protein combinations. So, we get 13 sets of SNPs in total. Each set of SNPs was then annotated with a flanking window of 1 kilobase (kb). We then used stratified LD score regression (S-LDSC) (23,39) to test whether each set of such SNPs was enriched for the heritability of each obesity-related complex trait. The Z-scores of each complex trait GWAS were harmonized by the munge_sumstats.py procedure of the ldsc software. LD scores of HapMap3 SNPs (MHC region excluded) for the annotated SNPs were computed using a 1-cM window (default). The heritability enrichment was evaluated by an enrichment score of the proportion of explained heritability divided by the proportion of annotated SNPs.

### Data availability

The summary statistics of 66 EV abundance phenotypes, corresponding to protein–protein combinations of 12 surface markers, will be made publicly available upon publication of this paper. Summary statistics of obesity-related GWAS are from the GIANT consortium: http://portals.broadinstitute.org/collaboration/giant/index.php/GIANT_consortium_data_files, URLs for GWAS we used in this paper are in Supplementary Material, Table S1. The full results of the EV-obesity associations are available in Supplementary Material, Tables S2 and S3, corresponding to Figures 3 and 4, respectively.

### Code availability

MultiABEL: https://github.com/xiashen/MultiABEL. PASCAL: https://www2.unil.ch/cbg/index.php?title=Pascal. LDSC: https://github.com/bulik/ldsc.

## Author Contributions

X.S., J.F.W. and D.W. initiated the study; X.S. coordinated the study; J.F.W. contributed ORCADES plasma samples; D.W. performed measurements using PBA; R.Z. performed data analysis; L.P. contributed to EV data pre-processing. Z.Y., T.L., Z.N. and Y.P. contributed to data analysis and interpretation; R.Z. and X.S. drafted the manuscript; All authors approved the final manuscript.

## Supplementary Material

EV_Manuscript_HMG_R1_SI_ddac069

Supplementary_Tables_HMG_rev1_ddac069
