# Supplementary material for: Genetic and phenotypic links between obesity and extracellular vesicles
Source: Hum Mol Genet. 2022 Mar 31;31(21):3643–51. doi: 10.1093/hmg/ddac069 (PMC9616576; doi:10.1093/hmg/ddac069)
Supplement: EV_Manuscript_HMG_R1_SI_ddac069 [file ev_manuscript_hmg_r1_si_ddac069.pdf]

Supplementary Information

for

Genetic and phenotypic links between obesity and extracellular vesicles

by Ranran Zhai, Lu Pan, Zhijian Yang, Ting Li, Zheng Ning,
Yudi Pawitan, James F. Wilson, Di Wu, Xia Shen

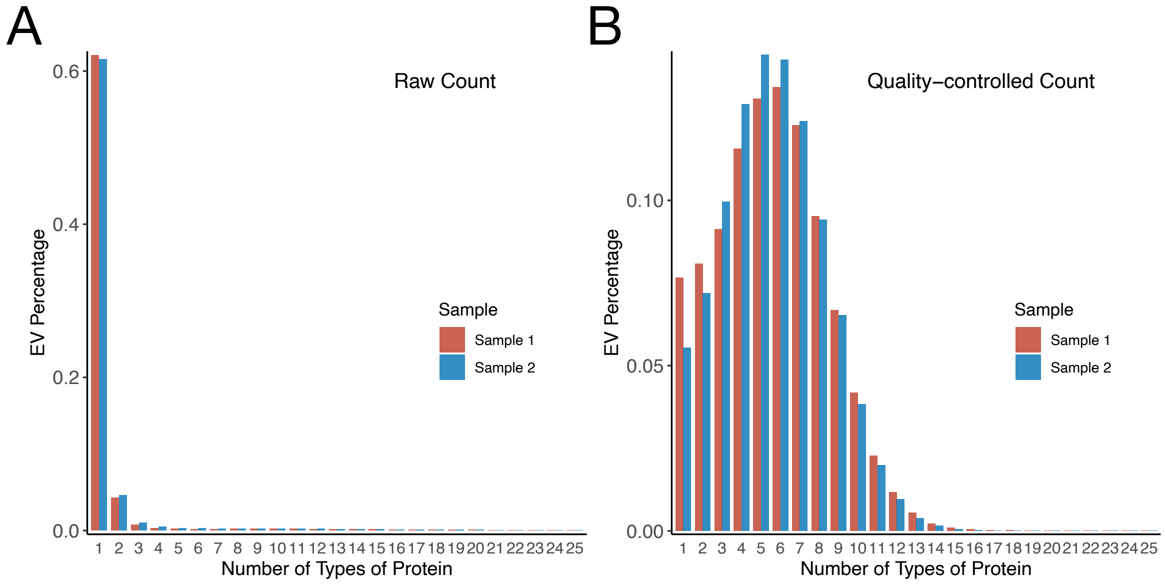

Supplementary Figure 1. Distribution of EV carrying different types of proteins. Histogram of EV that carry different number of types of proteins in 2 independent samples. **(A)** shows the raw count from original deep sequencing quantification data (50 million reads per sample), while **(B)** shows count after our data quality-control procedure (see **Methods**).

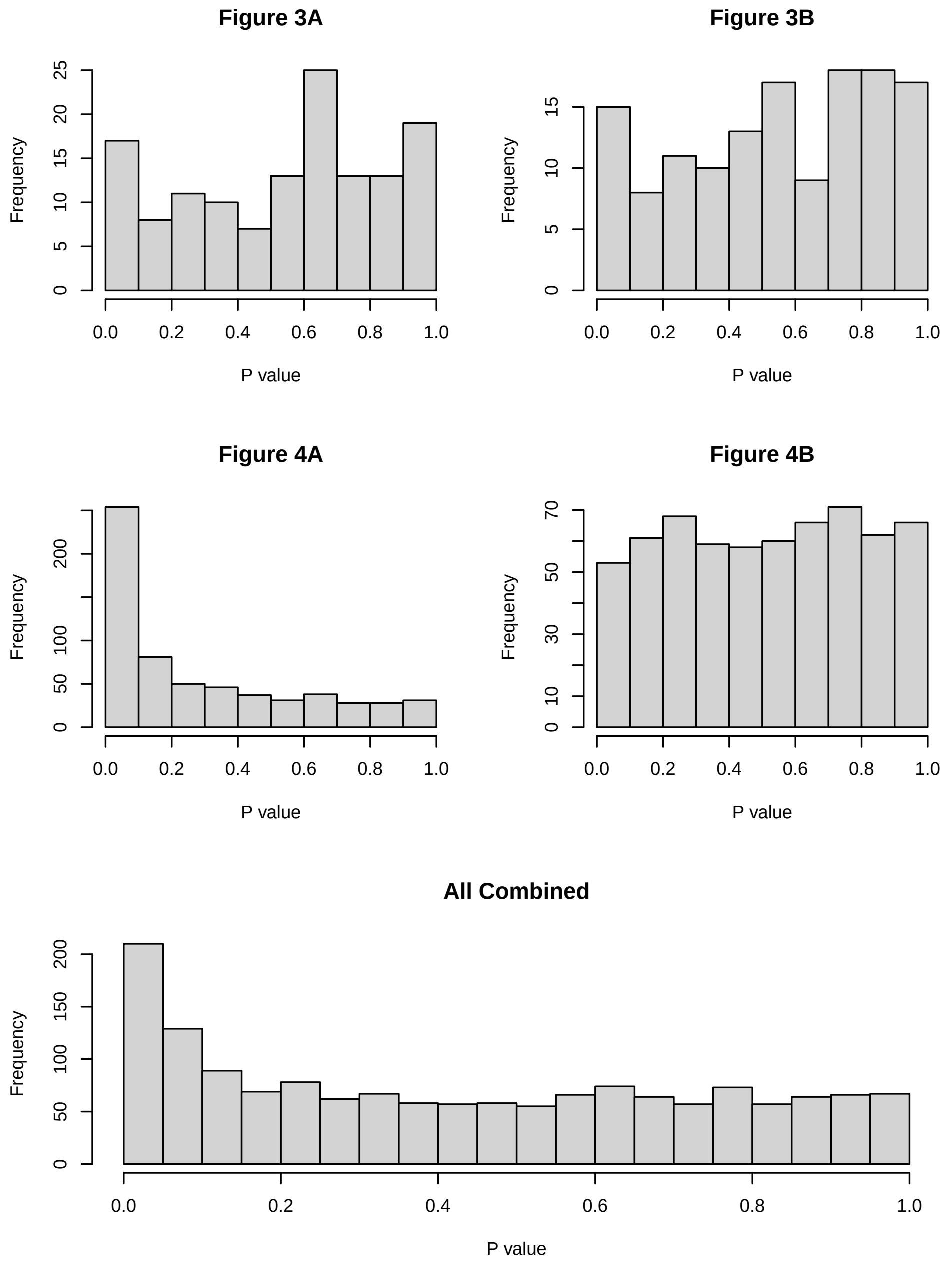

Supplementary Figure 2. P-value distributions of the associations between protein/EV levels and obesity. The histograms show the p-value distributions of the association tests in **Figures 3** & **4**. The top two rows correspond to the four panels of **Figures 3** & **4**. The bottom panel shows the combined p-value distribution.

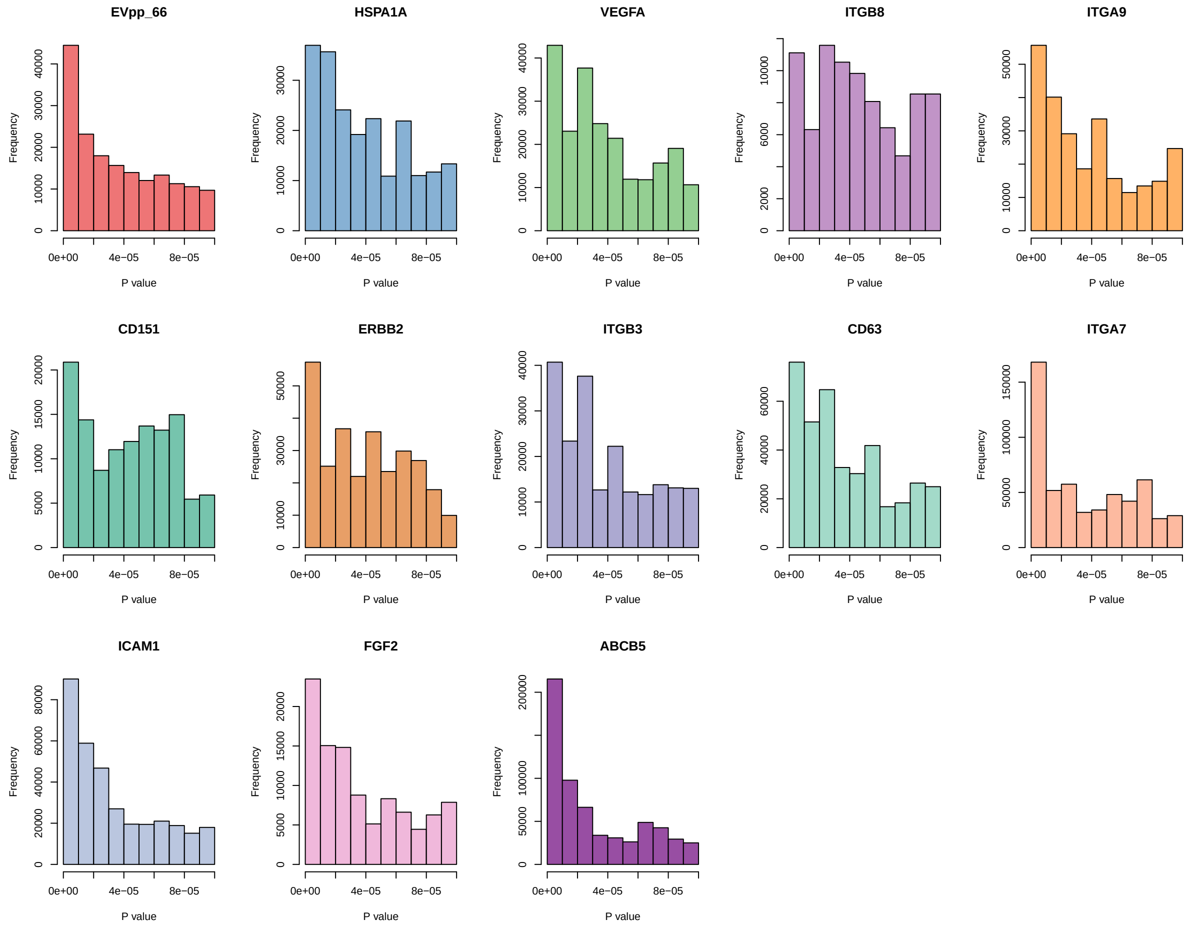

Supplementary Figure 3. Histogram of p-value distributions in the EV proteomic GWAS. The first sub-figure (named with EVpp_66) includes SNPs from 66 EV proteomic GWAS where EV carry two of 12 markers. The others include SNPs from 112 GWAS where EVs carry one of 12 markers, and each sub-figure was named with the corresponding marker. For example, the second sub-figure shows the p-value distribution of SNPs from 112 EV proteomic GWAS where EVs carry both HSPA1A and another marker from the other 112 proteins.

Supplementary Tables

Supplementary Table 1: GWAS summary statistics source for obesity-related phenotypes.

[See the Excel File]

Supplementary Table 2. Regression coefficients between the overall protein levels and anthropometric measurements in 96 ORCADES samples. For each trait (every 6 columns), the former 3 columns shows Estimate and standard error, R squared, P values of EV effect of all tested specific total protein levels on obesity related traits, named with Beta(SE), R-squared and P-value, respectively. The latter 3 columns shows Estimate and standard error, R squared, P values of EV-sex interaction effect of all tested total protein levels on obesity related traits, named with sex-Beta(SE), sex-R-squared and sex-P-value, respectively. For all columns, the first 5 rows were plasma total protein levels measured by PEA, left were total EV protein levels measured by PBA.

[See the Excel File]

Supplementary Table 3. Regression coefficients between the abundance of specific EVs and anthropometric measurements in 96 ORCADES samples. For each trait (every 6 columns), the former 3 columns shows Estimate and standard error, R squared, P values of EV effect of all tested specific EV levels on obesity related traits, named with Beta(SE), R-squared and P-value, respectively. The latter 3 columns shows Estimate and standard error, R squared, P values of EV-sex interaction effect of all tested specific EV levels on obesity related traits, named with sex-Beta(SE), sex-R-squared and sex-P-value, respectively.

[See the Excel File]
